# A Case of Metastatic Vulvar Choriocarcinoma Misdiagnosed as Vulvar Abscess: A Case Report

**DOI:** 10.1155/2024/9918452

**Published:** 2024-08-27

**Authors:** Alita Mrema, Prudence H. Kiwia, Shaban J. Shaban, Anwar Z. Mohamed, Latifa Rajab Abdallah, Rajabu Kiaratu, John Mahoyogo, Agapiti Chuwa, David H. Mvunta

**Affiliations:** ^1^ Department of Clinical Oncology Ocean Road Cancer Institute, Barack Obama Drive, P. O Box 3592, Dar es Salaam, Tanzania; ^2^ Department of Clinical Oncology Muhimbili University of Health and Allied Sciences, 9 United Nations Road, Upanga West, P. O Box 65001, Dar es Salaam, Tanzania; ^3^ Department of Surgical Oncology Ocean Road Cancer Institute (ORCI), P.O Box 3592 Barrack Obama Drive, Dar es Salaam, Tanzania; ^4^ Radiology and Imaging Section Ocean Road Cancer Institute, Barack Obama Drive, P. O Box 3592, Dar es Salaam, Tanzania; ^5^ Department of Physiology University of Dar es Salaam Mbeya College of Health and Allied Sciences, P. O Box 608, Dar es Salaam, Tanzania; ^6^ Department of Obstetrics and Gynecology Muhimbili University of Health and Allied Sciences, 9 United Nations Road, Upanga West, P. O Box 65001, Dar es Salaam, Tanzania; ^7^ Department of Obstetrics and Gynecology St. Joseph College of Health and Allied Sciences, P. O Box 11007, Dar es Salaam, Tanzania

**Keywords:** case report, choriocarcinoma, gestational trophoblastic disease, gestational trophoblastic neoplasia

## Abstract

**Background:** Metastatic vulvar choriocarcinoma, a rare ectopic gestational trophoblastic neoplasia (GTN), often presents a diagnostic challenge due to its mimicry of other conditions, particularly in resource-limited settings. Its primary symptom is abnormal vaginal bleeding without a clear cause. Consequently, diagnosing and managing it poses difficulties for many low-resource health facilities, as evidenced by the current case.

**Case Presentation:** We present the case of a 25-year-old, P2+2+2L2, who had a large painless, bleeding vulva mass for nearly 5 months. This followed a spontaneous abortion the month prior. The mass gradually increased in size and was accompanied by fever, pus discharge, and weight loss. Despite being treated at multiple health facilities for a vulvar abscess, there was no improvement. A diagnosis was finally made at a tertiary facility where elevated quantitative serum beta-human chorionic gonadotropin (hCG) (*β*-hCG) was noted. Due to uncontrollable vulva bleeding, she was referred to another tertiary facility for emergency radiotherapy. Following stabilization, chemotherapy was administered using the EMA-CO protocol.

**Conclusion:** The report highlights the difficulty in diagnosing vulvar choriocarcinoma, underscoring the importance of a high index of suspicion. Clinical tests such as serum (*β*-hCG) and imaging studies are crucial for diagnosis. In resource-limited settings, a simple strip-based urine pregnancy test with serial dilutions can be sufficient for diagnosing and managing vulvar choriocarcinoma.

## 1. Introduction

Gestational trophoblastic disease (GTD) encompasses a range of uncommon conditions resulting from an abnormal proliferation of the trophoblast of the placenta [1]. This spectrum includes benign and nonneoplastic lesions and malignant and neoplastic lesions [1]. Histologically, benign lesions comprise hydatidiform mole (partial/complete), exaggerated placental site (EPS), and placental site nodule (PST). Malignant lesions, such as invasive mole, choriocarcinoma, epithelioid trophoblastic tumor (ETT), and placental site trophoblastic tumor (PSTT), collectively form gestational trophoblastic neoplasia (GTN) [1]. Therefore, GTN is a subset of GTD that has developed malignant sequelae [1]. All of these lesions produce the pregnancy hormone human chorionic gonadotropin (hCG), except for PSTT, ETT, and PSN. hCG is an important biomarker of disease progression, response, and conducting posttreatment surveillance [1]. Consequently, with the emergence of newer therapies coupled with the utilization of this biomarker, cure rates have improved.

The reported incidence of choriocarcinoma in Southeast Asia and Japan is 9.2 and 3.3 per 40,000 pregnancies, respectively, higher than in the rest of the world [1]. In Africa, single studies in Egypt and South Africa have reported the incidence of GTN as 3.2/1000 live births and 0.5/1000 deliveries, respectively [2, 3]. Comparing worldwide incidence rates of choriocarcinoma is challenging due to the varying methods used to determine these rates [4]. Some authors report rates based on total pregnancies, deliveries, or live births [4]. Choriocarcinoma primarily manifests in the uterus but also occurs in multiple sites such as the fallopian tubes, ovary, lung, liver, spleen, kidneys, bowels, brain [1, 5], vagina, and vulva. Vulvar choriocarcinoma typically presents as a bulky tumor with hemorrhagic and necrotic areas [1]. Thus, it is commonly misdiagnosed as hematoma, vaginal tumor, or Bartholin abscess.

We present a woman found to have a large bleeding vulvar mass that presented a diagnostic dilemma and was confirmed only after an elevated serum quantitative beta-hCG (*β*-hCG).

## 2. Case Presentation

A 25-year-old woman, para 2+2, living 2, was admitted to the ward with a large bleeding vulva mass that had begun 5 months prior and progressively increased in size. Initially painless, it gradually became painful, prone to bleeding upon touch, and discharged pus. Subsequently, she developed fever, dizziness, headache, heartbeat awareness, easy fatiguability, and unintentional weight loss. There was no history of vaginal bleeding, vomiting, abdominal pain/distension, bone pain, lower limb pain, or loss of consciousness.

Prior to this condition, she had a history of spontaneous abortion a month earlier, where evacuation was performed, without any notable issues. She was then prescribed a short course of oral antibiotics.

During her illness, the patient underwent treatment at various health facilities for what was initially diagnosed as an abscess. Despite attempts at incision–drainage and administration of parenteral antibiotics, her condition did not improve. She was then referred to a tertiary referral facility, where the diagnosis of vulva choriocarcinoma with lung metastasis was established from elevated serum *β*-hCG. However, due to the severe bleeding (approx. 1.5 L) from the mass, she was referred to Ocean Road Cancer Institute (ORCI) for further management.

**Figure 1:**
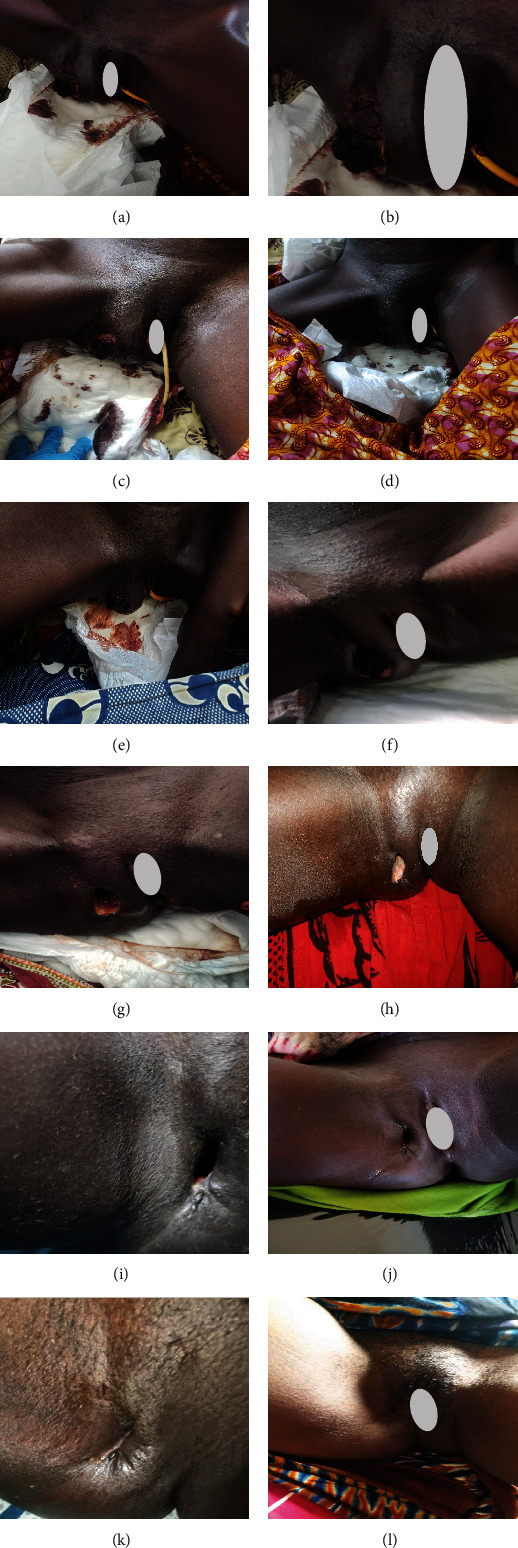
(a–l) Photographs showing various stages of treatment (before and after). (a–d) Macroscopic appearance of vulvar choriocarcinoma lesions before initiation of therapy. (e–k) Tumor shrinkage after the initiation of chemotherapy treatment. Chemotherapy administration in cycles: (e) 1st cycle, (f) 2nd cycle, (g) 3rd cycle, (h) 4th cycle, (i) 5th cycle, (j) 6th cycle, and (k) 7th cycle. (l) Current appearance 2 months after treatment completion.

**Figure 2:**
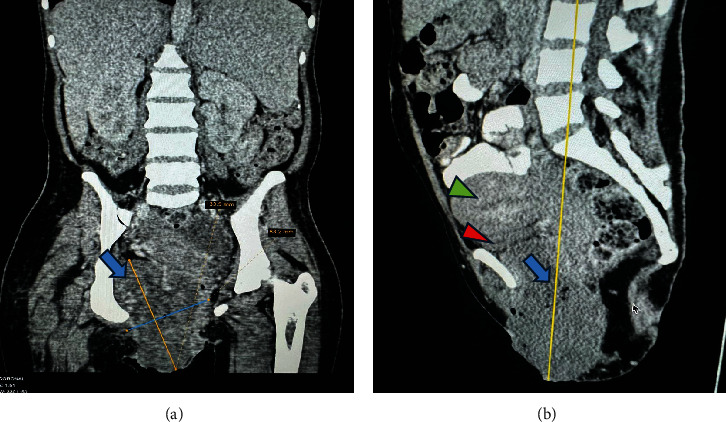
CT scan images showing a mass on the vulva. Reformatted images showing heterogenous enhancing mass on the right side of the vulva extending to the pelvis compressing the urinary bladder with no signs of infiltration and no bone involvement. (a) Coronal view, with a blue arrow showing the mass. (b) Sagittal view, showing mass (blue arrow), uterus (red arrowheads), and urinary bladder (green arrowheads).

**Figure 3:**
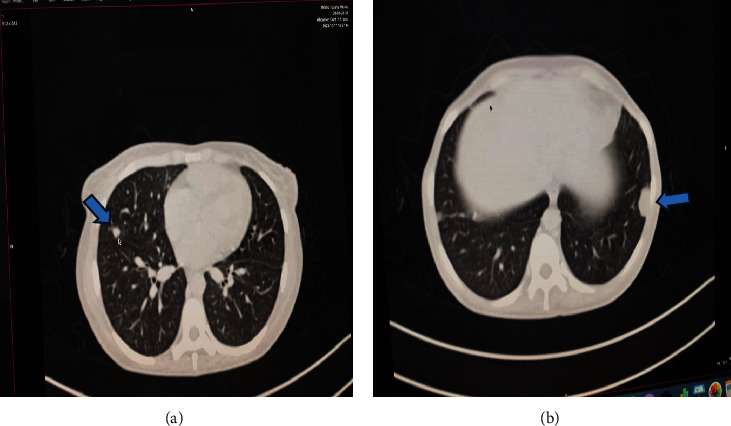
CT scan images showing lung nodules (cannon balls). Reformatted images show lung nodules on the lateral segment of the left lower lobe, (a) coronal view, with a blue arrow showing the lung nodule. (b) The sagittal view shows a pleural-based lung nodule (blue arrow).

Upon examination at ORCI, she presented with a vulvar mass approximately 10 × 8 × 6 cm, prone to bleeding upon touch, with necrotic tissues at the margins, and vaginal bleeding (Figures 1(a), 1(b), and 1(c)). Her initial serum hCG was 300,000 mIU/mL (Table 1). Imaging studies revealed a heterogenous enhanced mass originating from the right side of the vulva (Figures 2(a) and 2(b)), with an extension to the pelvic region ipsilaterally with an area of necrosis measuring 133.5 × 83.5 mm (Figures 2(a) and 2(b)). CT scan showed three nodular opacities (cannon balls), likely representing lung metastasis (Figure 3). Blood tests showed elevated white blood cells (19.25 × 10^9^/L), predominantly neutrophils (83.7%), and low hemoglobin (5.5 g/dL). The other biochemical tests such as serum creatinine, urea, AST, and ALT were unremarkable within normal ranges. She was classified as FIGO Stage III, due to extension to the lungs and genital tract involvement, with a WHO prognostic score of 8 (high risk), derived from (age < 40 = 0, abortion = 1, interval from index pregnancy = 0, pretreatment hCG = 2, largest tumor size = 2, site of metastases = 2, number of metastases = 1, and failed chemotherapy = 0).

Before treatment planning, she received four units of packed red blood cells and IV antibiotics (ceftriaxone and metronidazole for 5 days). Emergency radiation at 8 Gy in a single fraction was initiated to control bleeding followed by chemotherapy with EMA-CO (etoposide (E) 100 mg on Days 1 and 2, methotrexate (M) 100 mg on Day 1, actinomycin (A) 0.5 mg on Days 1 and 2, cyclophosphamide (C) 800 mg on Day 8, and leucovorin (O) 15 mg on Days 2 and 3). She received this regimen due to her WHO risk stratification as high risk (score 8) and is recommended by the National Comprehensive Cancer Network (NCCN) guidelines. The regimen EMA was administered alternately with CO weekly for seven cycles.

This regimen resulted in tumor shrinkage, wound healing (Figures 1(e), 1(f), 1(g), 1(h), 1(i), 1(j), and 1(k)), and improved patient condition. Additionally, this was accompanied by a serial drop of *β*-hCG after each cycle of chemotherapy (Table 1). Ultimately, there was significant tumor size reduction (Figures 1(e), 1(f), 1(g), 1(h), 1(i), 1(j), and 1(k)) and wound healing (Figure 1(l)).

## 3. Discussion

Choriocarcinoma, a malignant form of GTD, generally arises in the uterine corpus of reproductive-age women with coincident or antecedent pregnancy [6]. The present case aligns with this pattern, as the patient presented 1 month after experiencing a spontaneous abortion. Most cases tend to occur in the uterine cervix, and choriocarcinoma rarely presents primarily extrauterine [1].

The clinical diagnosis of extrauterine choriocarcinoma poses a challenge as symptoms are often nonspecific [1]. Vaginal bleeding stands out as the most common presenting symptom, yet it frequently mirrors other more prevalent disease entities [1, 5]. This situation elucidates the diagnostic dilemma encountered in the present case, where initial health facilities misdiagnosed the condition as a vulvar abscess. Subsequently, due to the lack of improvement, the patient was referred to a higher level facility.

A wealth of literature has coined a criterion for diagnosing this entity: absence of disease in the uterine cavity, pathologic confirmation of the diagnosis, exclusion of molar pregnancy, and exclusion of a coexistent normal intrauterine pregnancy [1, 6]. While several cases of uterine choriocarcinoma with metastasis to the vulva or vagina have been documented [5, 6], only a few cases of metastatic vulvar choriocarcinoma have been reported in the literature [7]. Metastatic vulvar choriocarcinoma has been frequently misdiagnosed as ectopic pregnancy [8], vaginal tumor [7, 9], dysfunctional uterine bleeding [9], and cervical polyp [10].

This can often result in a delay in proper management, a situation akin to the present case report. Since trophoblastic neoplasms such as choriocarcinoma produce an excessive amount of *β*-hCG, serial monitoring of its trend is highly useful for diagnosis, posttreatment follow-up, and response assessment [1, 7]. The mainstay of treatment is chemotherapy, as choriocarcinoma is highly chemosensitive [1].

Although treatment of choriocarcinoma is chemotherapy, surgery [7] and radiotherapy may sometimes be included [11]. The role of radiotherapy is very limited in choriocarcinoma and is mostly applied in metastatic disease [12]. For instance, in brain metastasis, it is given in the form of whole brain irradiation (WBRT) with hippocampal sparing technique or stereotactic radiosurgery (SRS) combined with multiagent chemotherapy or intrathecal administration of methotrexate [13]. Radiotherapy may also be utilized in the minor pelvic region to address residual disease [13] or tumors that cannot be surgically removed, thereby alleviating recurrent bleeding [13].

Diagnosing vulvar choriocarcinoma requires a high index of suspicion. Once suspected based on the aforementioned signs, perform a quantitative serum *β*-hCG test. Additionally, supportive investigations like ultrasonography or MRI of the pelvis, chest X-ray, or CT scan, if available, should be conducted to rule out lung metastasis [7]. Moreover, in low-resource settings, where quantitative serum *β*-hCG is unavailable, the use of a serial-strip urine pregnancy test in dilutions has been recommended as an effective screening tool for diagnosis [14].

Based on the presently described case, a thorough clinical history and a simple urine pregnancy test with serial dilutions should have been conducted to indicate the possibility of GTD, ultimately leading to a diagnosis of choriocarcinoma [14]. Serial dilutions are essential to prevent the antigen-excess phenomenon (hook effect), which occurs when high concentrations of antibodies or antigens hinder the formation of immune complexes, impairing their effectiveness [14]. Additionally, proper imaging techniques, preferably ultrasound or MRI, would suffice diagnosis and hence proper management.

## 4. Conclusions

Based on the case described, maintaining a high index of suspicion is paramount for diagnosing vulvar choriocarcinoma. Once suspected, perform serum *β*-hCG and imaging studies.

## Figures and Tables

**Table 1:** The pre- and postsurveillance quantitative *β*-hCG values. A sequential drop in *β*-hCG throughout treatment.

**Dates**	**Chemotherapy cycles**	**Quantitative *β*-hCG (mIU/mL)**
Sept. 2023	On admission	300,000
Oct. 19, 2023	1st	1840
Nov. 3, 2023	2nd	417
Nov. 27, 2023	3rd	84.49
Dec. 18, 2023	4th	10.62
Jan. 19, 2024	5th	4.57
Feb. 21, 2024	6th	5.80
April 3, 2024	7th	2.08

## Data Availability

The data that support the findings of this study are available on request from the corresponding author. The data are not publicly available due to privacy or ethical restrictions.

## Nomenclature

CT scan: computerized tomography scan
EMA-CO: etoposide, methotrexate, actinomycin D, cyclophosphamide, and oncovorin
ETT: epithelioid trophoblastic tumor
GTD: gestational trophoblastic disease
GTN: gestational trophoblastic neoplasia
hCG: human chorionic gonadotropin
MRI: magnetic resonance imaging
NCCN: National Comprehensive Cancer Network
PSTT: placental site trophoblastic tumor
SRS: stereotactic radiosurgery
WBRT: whole brain irradiation
